# Biochemical Mechanisms That Buffer the Effects of High Temperatures in the Sand-Dwelling Lizard *Holbrookia propinqua*

**DOI:** 10.3390/ani15050656

**Published:** 2025-02-24

**Authors:** Yessica Caballero Vázquez, Ahiezer Rodríguez-Tobón, Fausto Roberto Méndez de la Cruz, Edith Arenas-Ríos

**Affiliations:** 1 Maestría en Biología de la Reproducción Animal, Departamento de Biología de la Reproducción, Universidad Autónoma Metropolitana, Iztapalapa 09340, Mexico; yessica.caballero5@gmail.com; 2 Laboratorio de Morfofisiología y Bioquímica del Espermatozoide, Departamento de Biología de la Reproducción, Universidad Autónoma Metropolitana, Iztapalapa 09340, Mexico; ahiezerrod@yahoo.com.mx; 3 Laboratorio de Herpetología, Instituto de Biología, Universidad Nacional Autónoma de México, México City 04510, Mexico; faustor@ib.unam.mx

**Keywords:** desert lizard, oxidative stress, redox homeostasis, heat stress, TRPV1, spermatozoa

## Abstract

Reptiles, as ectothermic organisms, rely on environmental temperatures for optimal physiological performance. The thermal requirements for optimal locomotion in reptiles can severely affect their reproduction. However, some species are successful in environments with temperatures exceeding 40 °C. *Holbrookuia propinqua* is a species that reproduces at high temperatures, a difference from most species in the family *Phrynosomatidae*, to which it belongs. Adult male *H. propinqua* specimens were collected at their reproductive stage, kept in separate terrariums, and divided into four groups that were exposed to different temperatures. After seven days of exposure, both the testes and epididymides were removed. The organs were processed for immunohistochemistry and to determine SOD, CAT, and GPX activity. A sperm sample was obtained from the vas deferens. The GSI decreased with temperature, and the EI presented its maximum value at 24 °C. Sperm viability was lower at the highest temperature, and motility at 28 and 32 °C exceeded 90%. The specific SOD activity in the testis at 41 °C decreased by ~75%. In the epididymis, SOD activity decreased at 28 and 32 °C. Transient Receptor Potential Vanilloid 1 (TRPV1) increased after 28 °C at the interstitial level and increased almost twofold in the seminiferous tubules.

## Simple Summary

The sand-dwelling lizard (*Holbrookia propinqua*) is an important model for the study of the factors that allow its reproduction in extremely hot environments. This study addresses the physiological mechanisms that buffer the effects of high temperatures in a desert lizard, whereas those temperatures inhibit the fertility of non-thermophilic species.

## 1. Introduction

Reptiles, as ectothermic organisms, depend on environmental temperature to optimally perform their physiological and reproductive functions [1,2,3]. However, the reproductive capacity of males is favored by temperatures lower than those preferred, which in mammals is equivalent to body temperature, which decreases their fertility [4]. Recent studies have shown that the exposure of lizards to their preferred temperatures has detrimental effects on sperm production. As shown in lizards exposed over seven days, the cells undergoing spermatogenesis in the seminiferous tubules, before becoming sperm in the epididymides, showed damage, including abnormalities in sperm morphology, lower sperm concentrations, and reductions in sperm motility and viability.

It has been reported that, in mammals, the functions of the testis and epididymis are carried out at least 2–4 °C below body temperature [5]. If the temperature increases, it can affect testicular function [6,7] and interrupt spermatogenesis, which can generate giant, multinucleated cells [8], blocking the action of gonadotropins within the seminiferous tubules, which decreases testosterone synthesis [9] and alters the epithelial cycles. At the same time, it can interfere with the antioxidant capacity of the cells [10], generating oxidative stress (OS) and causing direct damage to the spermatozoa [11]. OS is generated by the imbalance between reactive oxygen species (ROS) and the participation of enzymes against oxidation damage. ROS regulation is maintained by reducing enzymes that keep them at adequate physiological levels. The primary defense system is formed by the following antioxidant enzymes: SOD, GPX, and CAT, which together eliminate O_2_^.−^ and H_2_O_2_ [12,13]. On the other hand, the presence of ion channels that are sensitive to elevated temperatures has been described, such as Transient Receptor Potential Vanilloid 1 (TRPV1), which plays an important role in the perception of thermal stress, protecting germ cells when they are exposed to harmful heat [14]. TRPV1 is a channel that serves as a multimodal detector of noxious heat and proton stimuli that depolarizes cell membranes and mediates Ca^2+^ and Na^+^ flux in response to temperatures above 43 °C and can be activated by capsaicin and inhibited by capsazepine [15]. TRP channels are found in the sperm of some mammals and regulate some functions such as motility, hyperactivation, capacitation, and acrosome reactions [16]. TRPV1 is crucial in the induction process of apoptosis. At the level of the male gonads, capsaicin induces apoptosis in Leydig cells and capsazepine suppresses them [14,17]. Additionally, it has been found in the starfish *Patiria pectinifera* that the early exposure of larvae to high temperatures triggers mechanisms of TRPA ion channels that allow organisms to be exposed to high temperatures that normally reflect lethal limits [18].

All those mechanisms may influence the distribution of the species, as some lizards cannot inhabit warm environments [4], whereas others may behaviorally control the increase in temperatures [19]. However, the present study addresses the mechanisms of a desert lizard. Even though some studies suggest that critical thermal limits are not related to environmental temperatures and that the relationship between some thermal parameters of lizards and the temperatures of their habitats is uncertain [20], more physiological and biochemistry studies are required to fully understand the limitations or attributes that explain the distribution of these lizards.

The earless lizard (*Holbrookia propinqua*, *Phrynosomatidae*), is a medium-sized, sand-dwelling organism (4.98–6.0 cm snout-vent length). *H. propinqua* presents with a synchronous seasonal polyestrian reproductive pattern, with mating happening between June and August [21]. It is distributed from the south of Texas, USA, to Veracruz through Tamaulipas, Mexico (with a discontinuous distribution) in loose sands, coastal beach dunes, peninsulas, and barrier islands. Altitudinally, it is located from 0 to 300 m [22,23]. It inhabits warm-arid to sub-humid climates with an average annual temperature of 24.31 °C (minimum–maximum: 14.15–32.58 °C), with an average annual precipitation of 99.2 mm. Their body temperature ranges between 31 and 43.1 °C, with 37 °C being their preferred temperature [24]. That is why they are classified as thermophilic organisms.

Some reports indicate that the thermal requirements for the locomotion of reptiles are above the optimal temperature required for reproduction [4]. In reptiles, as in mammals, there is a decrease in semen parameters when the optimal temperature increases [4]. However, more research is required to understand the effect of temperature on sperm parameters, particularly in reptiles [25,26]. Thermal stress generates ROS, leading to reproductive physiology problems [27]. Therefore, it is important to study a species of lizard that lives in an environment where the temperature exceeds 40 °C, such as *Holbroquia propinqua*, and reproduces at higher temperatures than most of the species from the *Phrynosomatidae* family. Therefore, sand-dwelling lizards are important models for the factor studies that allow their reproduction in a warm environment, whilst other related species were severely affected and even could be killed, especially developing embryos of viviparous species [28]. This study addresses the physiological mechanisms that buffer the effects of high temperatures that could be limiting for non-thermophilic species.

## 2. Materials and Methods

### 2.1. Capture of Organisms

A total of 25 adult males of *Holbrookia propinqua* in the reproductive stage were collected from June 18 to 21 in Playa Escondida, Tamaulipas (22°31′43.8″ N, 97°81′43.9″ W). The government authorization number was SGPA/DGVS/00962/22, granted by the Secretaría de Medio Ambiente y Recursos Naturales (SEMARNAT). This means that the species is not at risk, as it is not listed in NOM059 SEMARNAT-2010.

All lizards analyzed were adult males with a snout–vent length of 4.98–6.0 cm, demonstrating the characteristic coloration of mature males [22]. Once captured by the control group, the specimens were transferred in pet-type containers to the Sperm Morphophysiology and Biochemistry Laboratory at the facilities of Metropolitan Autonomous University-Iztapalapa.

### 2.2. Thermal Treatment Selection

The organisms that would be part of the treatments were transported to a laboratory and maintained in separate terraria with peat moss as a substrate, controlled 8 h photoperiods matching the capture locality, and 30–40% humidity. We fed individuals every other day with 2–3 *Acheta domestica* per individual, supplemented with calcium, and provided water ad libitum. Subsequently, the specimens were divided into 4 groups of 5 individuals each. The 4 groups with treatment were placed in Hova Bator 1602 N incubators where they were maintained at the required temperatures (28°, 32°, 37°, and 41 °C) for seven days during the lizards’ activity schedule, letting them rest at 1900 h to simulate the rest–activity period at 13 °C under normal conditions for this group, according to the schedules reported by [22,29].

The control group was euthanized by decapitation (a procedure approved by the Metropolitan Autonomous University–Iztapalapa bioethics committee) immediately upon arrival from the field and the groups subjected to the different temperatures were euthanized 7 days later, with sufficient exposure time to see adverse effects on reproduction [4], based on the guide for the care and use of laboratory animals (Institute of Laboratory Animal Research, 2010).

### 2.3. Obtaining Biological Material by Histology and TRPV1 Immunolocalization

Both testicles and epididymides were removed to determine the morphometric parameters, and the gonadosomatic index was calculated with the following formula: GSI = ((Testicular weight × 100)/Bodyweight), and in the same way for the epididymis: ESI = ((Epididymal weight × 100)/Bodyweight).

Only the organs on the left side were processed for histology. The epididymis was regionalized into *caput*, *corpus*, and *cauda*. The testicle was divided into two equal parts. Each section of the organs was placed in 10% formalin until its inclusion in paraffin. Subsequently, 5 µm sections were made with a rotating microtome to later carry out the staining with hematoxyline–eosine [30]. Finally, the slides were covered with Entellan and observed under a bright field microscope.

The cuts were deparaffinized at 60 °C for 30 min for the localization of TRPV1, permeabilized with 2% Triton X-100 for 10′ at room temperature, and subsequently blocked with 10% BSA in PBS for 60′ at room temperature, and then the samples were incubated with rabbit polyclonal Anti-TRPV1 primary antibody (Invitrogen by Thermo Fisher Scientific, Waltham, MA, USA) (1:200 in PBS) in a humid chamber at 4 °C in darkness for 24 h. Subsequently, 3 washes were performed with PBS and samples were incubated with the secondary antibody conjugated with Alexa Fluor 488 IgG (Invitrogen by Thermo Fisher Scientific, Waltham, MA, USA) (1:400 in PBS) for 90 min. Finally, 3 washes with PBS-Tween were performed to mount the sample with PBS-glycerol (1:1) [16] and samples were observed with a VELAB brand epifluorescence microscope model VE-146YT. The ImageJ 1.52 program was used to quantify the fluorescence index (FI) in 5 regions of the testis (interstice and seminiferous tubules) of 5 organisms per treatment (25 data points per individual) [31].

### 2.4. Determination of Enzymatic Activity

The organs on the right side (processed in the same way as the organs on the left side) were used to determine the enzymatic activity; they were placed in 1.5 mL Eppendorf tubes and introduced into liquid nitrogen for freezing and subsequent determination of enzymatic activity according to what was described by Arenas-Ríos et al. [12] and Campos-Rentería et al. [32].

The determination of the specific activity of SOD (EC.I.15.1.1) was carried out according to what was described by Arenas-Ríos et al. [12] and Campos-Rentería et al. [32]. Xanthine and xanthine oxidase (XOD) were used to generate superoxide radicals, which, upon reaction with 2-(4-iodophenyl)-3-(4-nitrophenol)-5-phenyl tetrazolium chloride (INT), formed red formazan dye. It was quantified spectrophotometrically at 505 nm (VWR). The decrease in chromogen due to the elimination of superoxide radicals by SOD activity was recorded.

GPX activity (EC.I.II.I.9) was determined by the Mills method [12,32]. GSH is a GPX substrate, and together with glutathione reductase (GPX/GR), it modulates the H_2_O_2_ threat, having water as a product; GSH is oxidized to GSSG disulfide, using NADPH as an electron donor. The readings were taken in the spectrophotometer at 340 nm (VWR), determining the decrease in NADPH.

GPX activity determination was conducted according to Arenas-Ríos et al. [12] and Campos-Rentería et al. [32]. First, a reaction system with KMnO_4_ was required, then the intensity of the color was recorded, and the color decreased due to peroxidation caused by H_2_O_2_ and potassium permanganate. The readings were carried out by reading in a spectrophotometer at 480 nm (VWR).

### 2.5. Analysis of Sperm Parameters

A sperm sample was obtained directly from the vas deferens and placed in a Tyrode physiological medium for subsequent evaluation [4,33] Motility was determined by placing 10 µL of the sperm sample on a slide and counting 100 cells under a bright field microscope with 400× magnification. A distinction was made between motile and immotile gametes. To determine the number of spermatozoa obtained from the vas deferens, the sample containing the spermatozoa was diluted with distilled water (1:1000); 10 µL was placed at each end of a Neubauer chamber, and 10 quadrants were counted [33]. Smears were made with 5 µL + 5 µL of eosin-nigrosine and left to dry at 36 °C. They were observed under a 400× bright field microscope and 100 spermatozoa were counted to differentiate between live and dead spermatozoa [33].

### 2.6. Statistical Analysis

For the normality and homoscedasticity determination, a Kolmogorov–Smirnov test and Levene test were performed [34]. When the data formed a Gaussian bell, parametric tests were carried out to compare between groups (more than 2), performing an ANOVA test followed by a Tukey–Kramer post hoc test, considering statistical differences when *p* < 0.05. If the variances did not present normality, a Kruskal–Wallis test was performed followed by a Dunn’s post hoc test.

## 3. Results

### 3.1. Gonadosomatic and Epididymal Index

**Figure 1:**
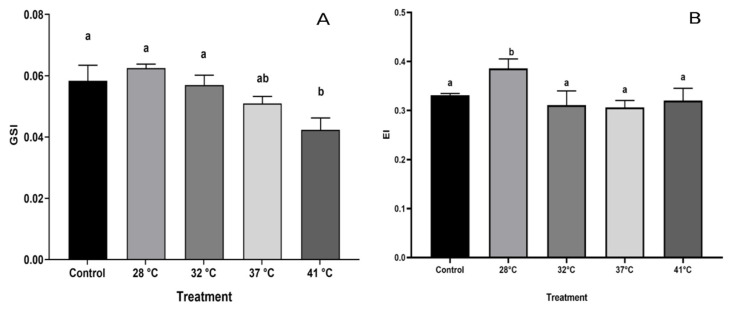
Gonadosomatic and Epididymosomatic Indexes of *Holbrookia propinqua* were obtained after the organisms were subjected to different temperatures. (**A**) Testicle; (**B**) epididymis. Bars represent mean ± SD. Different letters represent significant differences between treatments. ANOVA, Tukey–Kramer post hoc test (*p* < 0.05). *n* = 5.

The GSI showed variation when the organisms were subjected to different temperatures (F = 9.508, *p* = 1.168 × 10^−5^). The GSI of the control was 0.05% without significant changes in treatments below 32 °C. However, in the 37 °C treatment, a lower GSI occurred [0.01% (*p* < 0.05)], the GSI being even lower, in treatments up to 41 °C (Figure 1A). The epididymal index (EI) indicates that in the treatment at 24 °C, the epididymis presents its maximum value (0.4%) compared to the other temperatures (*p* < 0.05) (Figure 1B).

### 3.2. Effect of Temperature on Sperm Parameters

**Figure 2:**
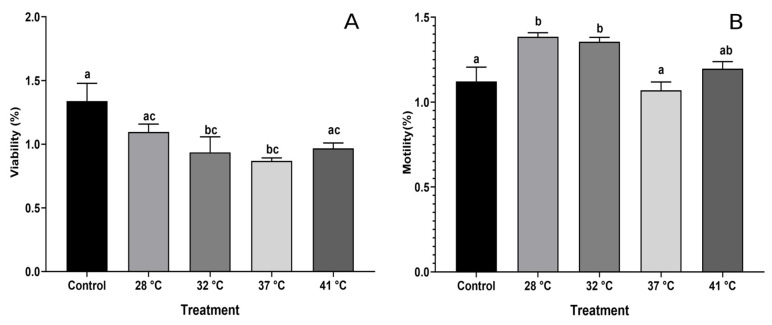
Sperm parameters (viability (**A**) and motility (**B**)) were obtained from the epididymis of *Holbrookia propinqua*. The percentage data were transformed to arco-sin ((x)1/2). Different letters represent significant differences between treatments (ANOVA, Tukey–Kramer post hoc test, *p* < 0.05).

Sperm viability was lower in the higher-temperature treatments, considering the control treatment and up to 32 °C (*p* < 0.05), resulting in a ~30% decrease in viability. However, sperm survival remained unchanged in treatments up to 41 °C (60%) (Figure 2A). In the case of sperm motility, it was observed that in treatments at temperatures of 28 and 32 °C, it exceeded 90% compared to the control (80%), resulting in similar motility in treatments at 37 and 41 °C (Figure 2B).

### 3.3. Enzymatic Activity

**Figure 3:**
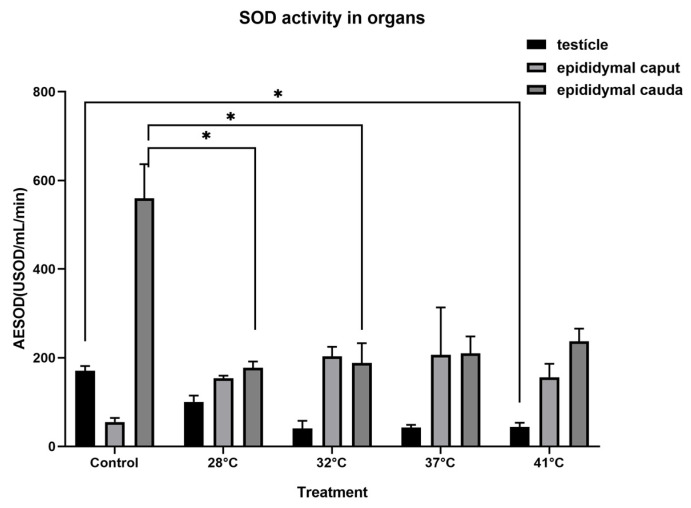
Superoxide Dismutase (SOD) determination is expressed as specific activity (USOD = amount of the enzyme that inhibits 50% of formazan) in the testicle (black bars) and epididymis (*caput*: light grey bar; *cauda*: dark grey bar) of *H. propinqua*. Bars represent averages ± SE. Asterisks denote significant differences when the same organ is compared between treatments (ANOVA, Tukey–Kramer post hoc test, *p* < 0.05). *n* = 5 organisms by treatment.

**Figure 4:**
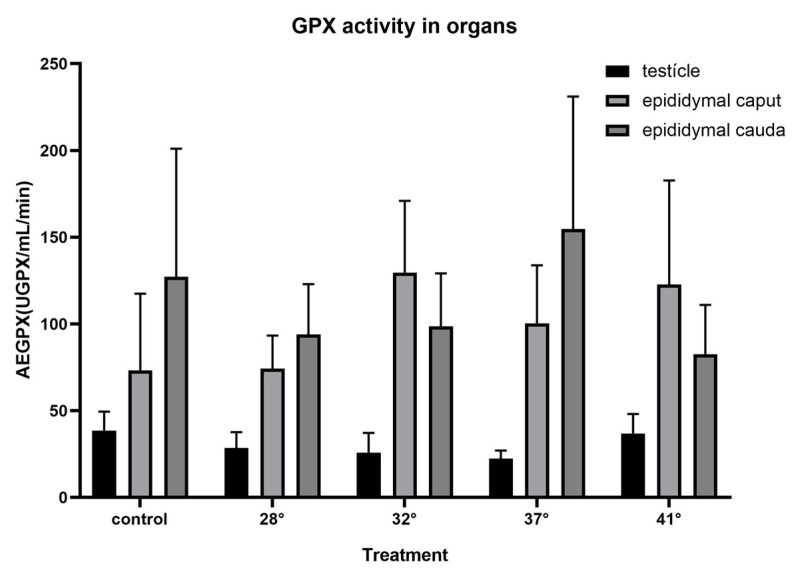
Glutathione Peroxidase (GPX) determination is expressed as specific activity (expressed as UGPX [nmol NADPH oxidized per min]) in the testicle and epididymis of *H. propinqua* subjected to different temperatures. *n* = 5 organisms by treatment.

**Figure 5:**
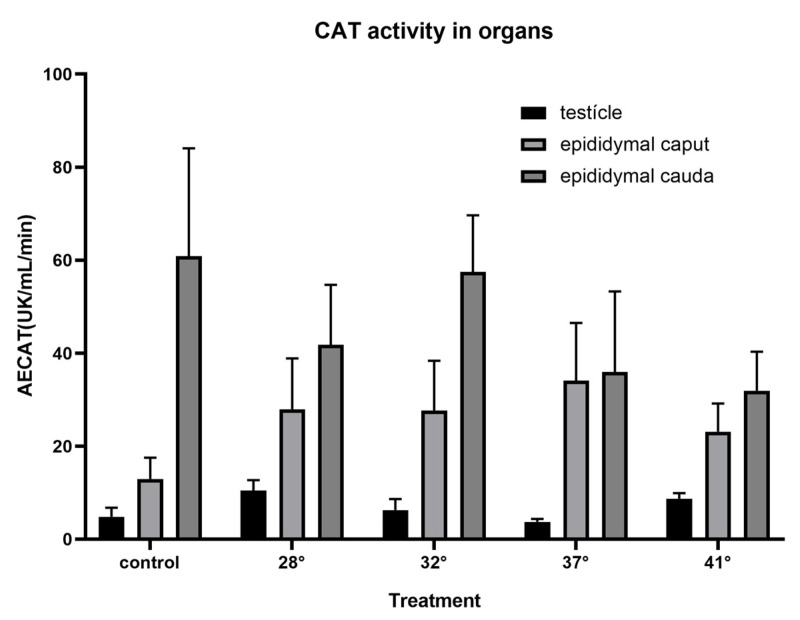
Catalase (CAT) determination is expressed as specific activity (expressed as K [constant rate of the first-order reaction]) in the testicle and epididymis of *H. propinqua* subjected to different temperatures. *n* = 5 organisms by treatment.

The specific SOD activity determined in the testis of *H. propinqua* at a temperature of 28° and up to 37 °C showed no differences. However, SOD activity in the 41 °C treatment compared to the control group (*p* = 0.0534) decreased by ~75% (Figure 3). In the epididymis, SOD activity decreased in the 28 and 32 °C treatments at the level of the epididymal cauda (F = 1.250, *p* = 0.3382; F = 3.894, *p* = 0.0215, respectively), compared to the caudal region of the control group (Figure 3). It can be seen in Figure 4 that there are no significant differences when comparing GPX activity in the testis (F = 0.5336, *p* = 0.7127) and the different regions of the epididymis (*caput*, *p* = 0.625; *cauda*, F = 0.3097, *p* = 0.8681) between treatments. Likewise, no significant differences were found in testicular or epididymal CAT activity in the various treatments to which the organisms were subjected (testis, F = 0.7122, *p* = 0.5932; *caput*, *p* = 0.6296; *cauda*, *p* = 0.7396) (Figure 5).

### 3.4. Histology and TRPV1 Immunolocalization

**Figure 6:**
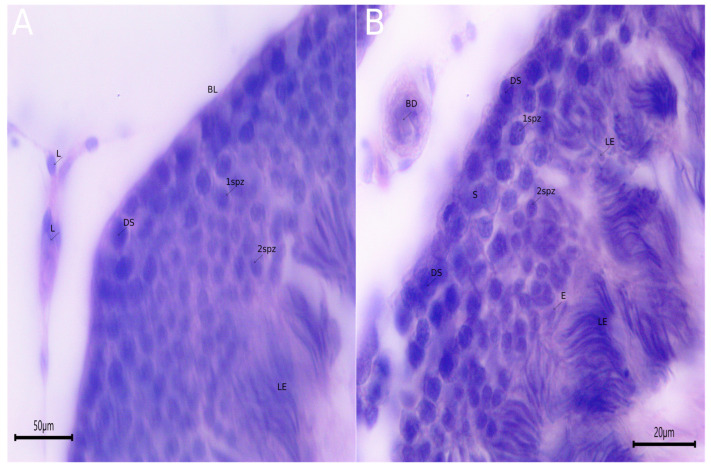
Micrographs of testis of *H. propinqua*. (**A**) Leydig cells (L); basal lamina (BL); dark spermatogonia (DS); primary spermatocytes (1spz); secondary spermatocytes (2spz); and elongated spermatids (LE). (**B**) Sertoli cell (S); blood vessels (BD); spermatids (E). Hematoxylin–eosin (5 µm), 400×.

To have a better understanding of the presence of TRPV1 channels in the different cells that make up the testicular tissue, a histological description of the testis of *H. propinqua* was carried out, in which the following can be identified: Leydig cells (L); basal lamina (BL); blood vessels (BD); Sertoli cell (S); dark spermatogonia (DS); primary spermatocytes (1spz); secondary spermatocytes (2spz); spermatids (E); and elongated spermatids (LE) (Figure 6A,B).

**Figure 7:**
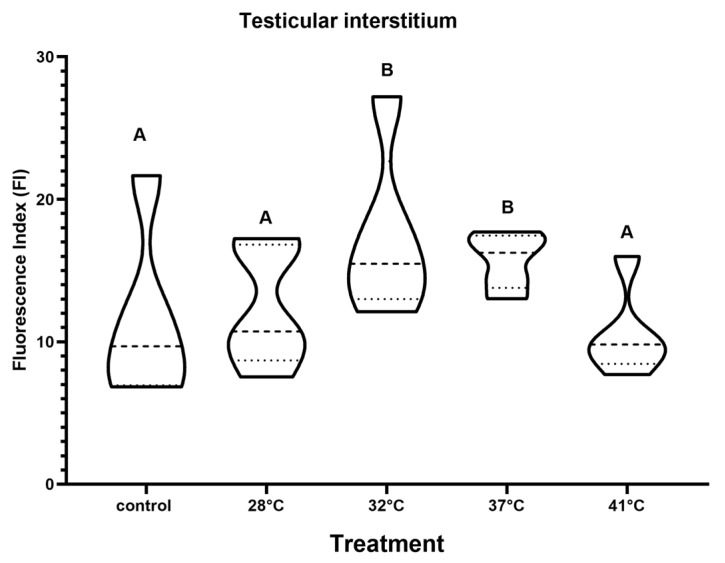
TRPV1 expression in the testicular interstitium of *H. propinqua* expressed as fluorescence index (FI) in the different treatments. Different letters represent significant differences between treatments (ANOVA, Tukey–Kramer post hoc test, *p* < 0.05). *n* = 5 organisms by treatment.

**Figure 8:**
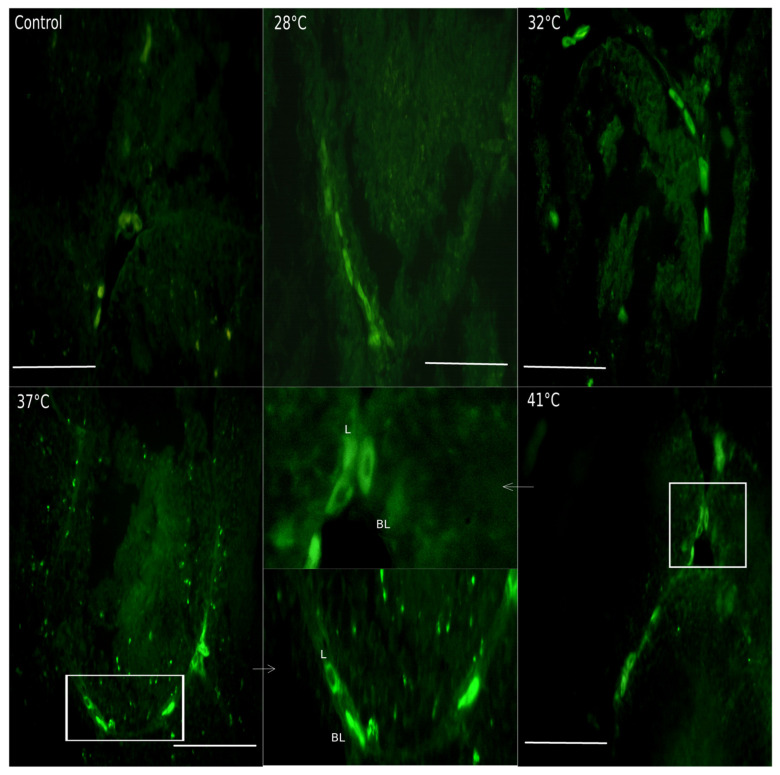
TRPV1 immunolocalization in the testicular interstitium of *H. propinqua* in the different treatments. TRPV1 increases after 28 °C; in the 32 and 37 °C treatments, it is observed in Leydig cells. A) Leydig cells (L); basal lamina (BL). The arrows point to what is indicated by the white box at each temperature. Fluorescence microscopy 400×. Bar = 100 μm.

**Figure 9:**
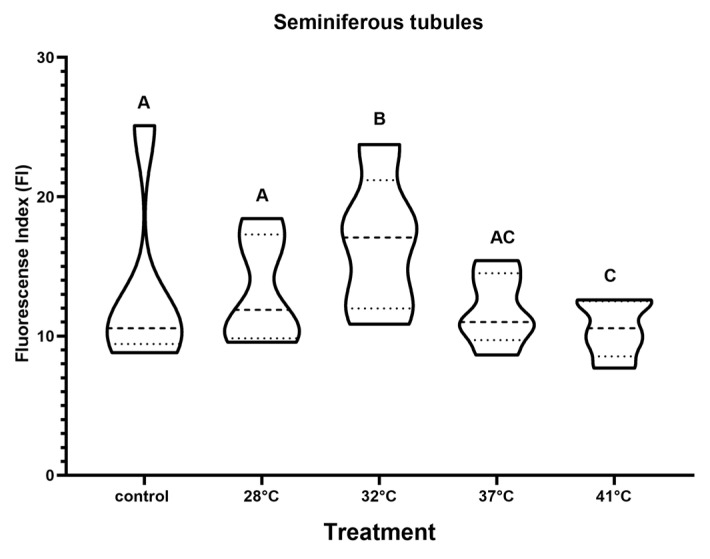
TRPV1 expression in the seminiferous tubules of *H. propinqua* expressed as fluorescence index (FI) in the different treatments. Different letters represent significant differences between treatments (ANOVA, Tukey–Kramer post hoc test, *p* < 0.05). *n* = 5 organisms by treatment.

**Figure 10:**
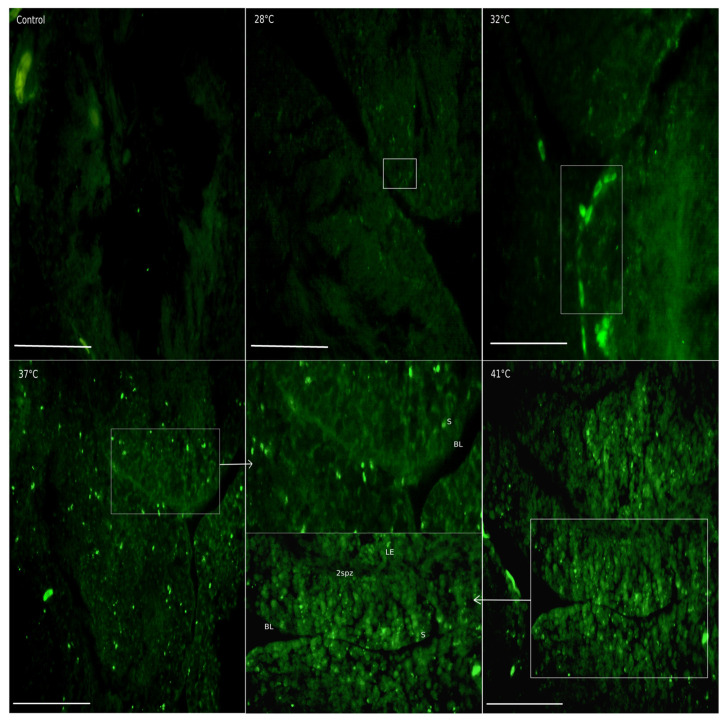
TRPV1 immunolocalization in the seminiferous tubules of *H. propinqua* in the different treatments. The white box indicates the places where the greatest expression of the channel was found. The presence of TRPV1 in the basal lamina of the seminiferous tubules becomes more evident at 32 °C. Marks against TRPV1 are observed in Sertoli cells at 37 °C, and in spermatogonia and spermatocytes as 41 °C is reached; its localization is evident in all the cellular strata that make up the seminiferous tubule. Basal lamina (BL); secondary spermatocytes (2spz); and (B) Sertoli cell (S). The arrows point to what is indicated by the white box at each temperature. Fluorescence microscopy 400×. Bar = 100 µm.

The presence of TRPV1 has been described in the testicular interstitium of some murine species [35]. In *H. propinqua,* TRPV1 increases after 28 °C at the interstitial level (Figure 7); in the 32 and 37 °C treatments, it is observed in Leydig cells that express TRPV1 (Figure 8), reaching values of FI = 15 and 16, respectively (*p* < 0.05) (Figure 7). As the temperature increases from 37 to 41 °C the entire interstitial space presents a marked signal against TRPV1 (Figure 8). However, the FI determined at 41 °C does not present differences compared to control (*p* < 0.05). At the level of the seminiferous tubules, we found that the presence of TRPV1 increases almost twice as much (FI = ~20) compared to the control (FI = 10) (Figure 9) during temperature increases; at 32 °C, the presence of TRPV1 in the basal lamina of the seminiferous tubules becomes more evident (Figure 10). At 37 °C, marks against TRPV1 are observed in Sertoli cells, spermatogonia, and spermatocytes; as 41 °C is reached, its localization is evident in all the cellular strata that make up the seminiferous tubule (Figure 10). However, the FI decreases to 37 (FI = 10) and 41 °C (FI = 10) (*p* < 0.05), making it evident that the highest concentration of TRPV1 is focused on the basal lamina and spermatogonia (Figure 9).

## 4. Discussion

There is a threat of rising temperatures because of climate change. It is predicted that 38% of desert amphibian and reptile populations will become extinct in the next 50 years, as lizards are exposed to higher environmental temperatures while exceeding their thermal limits [36]. The following adaptations have been proposed to cope with this problem: migration to more favorable thermal environments, with higher altitudes to reach lower temperatures; acclimatization (phenotypic plasticity). The inability to cope with these changes will lead to the extirpation of populations or extinction of species [36]. However, some species seem to be adapted to extremely high temperatures, such as the model addressed in the present work, and some antioxidant enzymes and TRPV channels could be involved to buffer the effects of the high temperatures. The advantages of thermophilic lizards in a warm environment are that they can benefit from temperature increases [37] and may expand their ranges. Therefore, the fact that lizards possess mechanisms that buffer the effects on the reproductive system of the increased temperatures may promote a broader distribution, which is a major issue that should be addressed in future studies.

Walsh et al. [38], using two fruit fly species as a model, mention that sperm resilience can vary between species. The finding is that mature sperm from *Z. indianus* are likely more resilient than sperm from *D. virilis*, which seems to be happening with *H. propinqua.*

Temperature is a determining factor in sperm development in scrotal mammals; spermatogenesis takes place 2 to 8 °C below body temperature [5,39,40]. Some reports indicate that increased temperature in the scrotal region results in a severe increase in programmed death in spermatogenic cells and the formation of multinucleated giant cells and vacuolization in seminiferous tubules after the onset of germ cell apoptosis [6,41,42]. The stress generated at the cellular level favors the increase in free radicals and ROS, including superoxide anion, hydrogen peroxide, and hydroxyl ion, which can cause male fertility problems [43]. Although apoptotic cell death plays an important role in the removal of debris cells [40,41], it is known that immersion of the testes in hot water baths at ~43 °C for 15–20 min results in apoptosis of germ cells (elongated spermatids and spermatocytes) [44], since it favors the increase in the pro-apoptotic factor Bax-xL and decreases the participation of Bcl [40,45]. If there were an imbalance between ROS production and the redox defense/repair system, what we know as OS, i.e., unsaturated fatty acid bonds, protein sulfhydryl bonds, protein sugars, and even cell DNA could be damaged [13]. The primary antioxidant defense system to regulate ROS production is formed by the enzymes SOD, GPX, and CAT, which help maintain ROS at physiological levels that are harmless to the cell [13,46,47,48].

The SOD enzyme uses two O_2_^.−^ molecules as a substrate, generating H_2_O_2_ as a product, a precursor molecule of OH−, the most dangerous radical, capable of causing oxidative damage in various biomolecules of the cell. However, GPX and CAT synergistically eliminate H_2_O_2_, since GPX is activated at low concentrations of H_2_O_2_, and when H_2_O_2_ increases, CAT is activated [12,13,32]. In *H. propinqua*, SOD activity decreases in the testis when the temperature reaches 41 °C. This decrease prevents the formation of H_2_O_2_, the only ROS that can cross cell membranes and the precursor of the most dangerous ROS (·OH), so the increase in SOD activity only favors the redox system if at least the GPX activity increases, thus regulating the cell’s redox system, as has been reported before in other species [12]. Therefore, the decrease in SOD activity could be responsible for the broad spectrum of thermal tolerance presented by *H. propinqua*, which ranges from 25 to 40 °C (Figure 3). This suggests that SOD is a molecular entity that acts as a protective agent as the testicular temperature increases in this organism, avoiding OS in the cells that make up the epithelial tissue of the testis.

Similarly, in the epididymis, SOD shows a decrease in its activity from 25 to 32 °C. This is evidence of the regulation of H_2_O_2_ production. This is more important if we consider that it is in the epididymis where sperm acquire their fertile capacity and are stored until copulation; as mentioned above and observed in the results (Figure 4 and Figure 5), there are no significant differences in the activity of GPX or CAT in the testicle and epididymis of *H. propinqua*, suggesting that the decrease in SOD activity is sufficient to maintain the REDOX balance necessary for the functioning of the cells in this species of lizard, since the increase in the activity of the CAT and GPX enzymes could mean an imbalance of the redox system, if we take into account that ROS have an important role in cell signaling, particularly in important processes for sperm physiology [12].

The above can be supported by the fact that *H. propinqua* epididymal sperm do not show a decrease in their motility when the temperature increases (Figure 2B). They maintain their mobility above 70% when reaching 41 °C; this implies that the redox system in the epididymal environment remains in balance when the gametes reach the distal region of the epididymis and remain stored. In addition to maintaining sperm functionality by preventing OS, it also maintains ROS levels at adequate levels, allowing the sperm to acquire the ability to move [49]. However, epididymal spermatozoa obtained from lizards exposed to 37 °C reduce their viability by 30% compared to the control group (Figure 2A). The results obtained at this temperature suggest that the TRPV1 ion channel could be participating in the protection of sperm, which is activated at temperatures above 37 °C. The above is an adaptation that allows *H. propinqua* to live and reproduce at 40 °C.

An acidic microenvironment is crucial for male fertility since the sperm cannot fertilize an egg without proper acidification of the acrosome [46]. They express a variety of ionic channels, including TRPV1, that play a crucial role during spermiogenesis [50,51]. TRPV1 leads to an influx of cations into the cell, resulting in depolarization, neuronal hyperexcitability, and ultimately the sensation of pain. This channel can be activated by noxious heat (>40 °C), low extracellular pH (<5.9), and a variety of chemical mediators [46,52,53]. In H. propinqua, TRPV1 expression decreases in the seminiferous tubules with increasing temperature (41 °C). The presence of TRPV1 is lower compared to lower temperatures, which would modify the flow of Na^+^ and Ca^2+^ ions into the cells, which intervene in the signaling pathways dependent on second messengers such as calcium, which are necessary during the formation of spermatozoa and their subsequent structural modification during spermiogenesis. The presence of TRPV1 from spermatogonia to spermatocyte, and even of Sertoli cells suggests its involvement in the temperature-dependent regulation of spermatogenesis [54,55,56].

It is important to consider that an acidic microenvironment is crucial for male fertility since the sperm cannot fertilize an egg without adequate acidification of the acrosome; therefore, an acidic environment is required in the testis, but could it be activating TRPV1? [46].

TRPV1 decreases at 41 °C in *H. propinqua* gonads (Figure 7 and Figure 9). Responses to changes in ambient temperature depend not only on the level of TRPV1 expression but also on TRPV1 activity. Vandewauw et al. [57] demonstrated that the TRPV1/TRPM3/TRPA1 trio needs to be knocked out to abolish heat sensing in mice. This implies that other compensatory pathways besides TRPV1 regulate temperature perception and thermoregulation. Wen et al. [58] mention that TRPV1 protein expression in brown fat and liver of gerbils was positively correlated with PKA expression. Other studies have also shown that TRPV1 activity is regulated by the PKA pathway [59]. The expression of TRPV1 and PKA in the brown fat of gerbils was positively correlated with the amount of time spent by gerbils under high or low temperatures, indicating that TRPV1 may participate in behavioral thermoregulation in gerbils through the PKA pathway [58].

Leydig cells are essential for synthesizing testosterone, which is necessary for maintaining spermatogenesis in the seminiferous tubules [60]. *H. popinqua* does not show a decrease in TRPV1 at the interstitium level, indicating that this channel could detect temperature changes, preventing damage to the Leydig cells and therefore an adequate synthesis of testosterone. *H. popinqua* does not show a decrease in TRPV1 at the interstitium level, indicating that this channel could be detecting temperature changes, preventing damage to Leydig cells and therefore an adequate synthesis of testosterone, since, in mammals, the increase in temperature generates cell death in germ cells and Leydig cells, causing male infertility [61,62].

Androstenedione activates TRPV1, dehydroepiandrosterone (DHEA) inhibits TRPV1 activation, and testosterone is less effective at inhibiting TRPV1 activation [63]. This could suggest that testosterone levels do not decrease, although this is a determination that could be made in the future. TRPV1 activation in male germ cells is linked to cell survival or death [54,64,65], as heat shock proteins control cell viability under various stress conditions [66,67].

Some studies have shown that TRPV1 KO mice are more susceptible to OS. However, it was found that in TRPV1 KO young mice, there is an extended lifespan and maintained youthfulness compared to WT at the same age; this indicates that TRPV1 may be inducing testicular apoptosis [35,65,68,69]. Higher expression of TRPV1 was found in testes of adult mice, in addition to its localization in Leydig cells in young mice [35,70,71].

Anandamide is a main component of endocannabinoids; it is involved in various physiological and pathological functions of organisms and has been shown to activate TRPV1 [46] by binding to the same site as capsaicin (8-methyl-N-vanillyl-6-nonenamide) [52], an active ingredient that accounts for the pungency of hot peppers [40,72]. In a mouse model, capsaicin proved to be a protective agent against apoptosis in the spermatogenic cells after scrotal hyperthermia [5,73].

Like endocannabinoids, other molecules such as ATP, ammonia, polyamides, spermine, putrescine, and protons can activate TRPV1. ATP was identified as a TRPV1-sensitizing molecule that binds to TRPV1 in a region between ankyrin repeats 1–3 [74]. Activators of PKC have been shown to sensitize TRPV1 channels to endogenous and exogenous agonists and reduce the temperature threshold for channel activation. The cytosolic domain of TRPV1 contains serine residues which could be phosphorylated by PKC [75,76]. Protons decrease the temperature threshold for TRPV1 activation such that even moderately acidic conditions (pH ≤ 5.9), such as hypoxia, ischemia, and inflammation, can activate the channel at room temperature [77,78]. Ammonia (NH_3_) activates TRPV1 (and TRPA1) in sensory neurons through a mechanism that involves a cytoplasmic histidine residue [78]. Polyamines such as spermine, spermidine, and putrescine, by their cationic charge, directly activate TRPV1 in a charge-dependent manner. The threshold for activation by spermine is rather high (~500 μM), but spermine can enhance the capsaicin evoked.

As happens in testicular hyperthermia during the inflammatory process, the participation of TRPV1 has been implicated. Prostaglandins and ATP, among other molecules, sensitize TRPV1 through the mobilization of Ca^2+^ and PKC, reducing the threshold of response to temperature, and a certain concentration of protons is necessary for TRPV1 activation [52]. Finally, it has been suggested that TRPV1 can form heterodimers with TRPV3 to exhibit increased temperature sensitivity and a lower threshold for activation and sensitization by heat compared to the monomeric receptors [52,79].

## 5. Conclusions

Thermophilic reptiles show fundamental adaptations to inhabit desert or sandy hot areas. This work shows the presence of biochemical mechanisms within the testes to protect sperm during the reproductive season. In contrast with nonthermophilic lizards that are decreasing in their areas of distribution or going extinct, the physiology of those mechanisms in sand-dwelling or desert lizards will promote the expansion of their distribution. Therefore, it is mandatory to conduct more studies that explore in detail the relationship between testosterone and TRPV1, the possible signaling pathways that involve this channel in apoptosis, and their effects on the benefits to species in the face of climate change.

## Data Availability

Dataset available on request from the authors.
